# Exceptional irradiation and corrosion resistances of a TaC nanoceramic coating deposited onto ZIRLO™ fuel cladding

**DOI:** 10.1038/s41529-026-00813-9

**Published:** 2026-05-29

**Authors:** Stefan Fritze, Barbara Osinger, Petter Ström, Graeme Greaves, Per Eklund, Oscar M. Prada-Ramirez, Vladimir M. Vishnyakov, Matheus A. Tunes

**Affiliations:** 1 Department of Chemistry – Ångström Laboratory, Uppsala University, Uppsala, Sweden; 2 Department of Physics and Astronomy, Materials Physics, Uppsala University, Uppsala, Sweden; 3 United Kingdom Atomic Energy Authority, Culham Campus, Abingdon, UK; 4 Departamento de Engenharia Metalúrgica e de Materiais, Escola Politécnica da Universidade de São Paulo, São Paulo, Brazil; 5 Inomorph Ltd., 3M Buckley Innovation Centre, Huddersfield, UK; 6 Department Metallurgy, Chair of Non-Ferrous Metallurgy, Montanuniversität Leoben, Leoben, Austria

**Keywords:** Energy science and technology, Materials science

## Abstract

Improving the accident tolerance of zirconium-based nuclear fuel claddings is essential to mitigate oxidation-driven hydrogen generation under loss-of-coolant conditions. Here, a nanostructured tantalum carbide (TaC) ceramic coating is deposited onto ZIRLO™ fuel cladding by non-reactive DC magnetron sputtering. The coating was systematically assessed with respect to its microstructural stability, irradiation tolerance, and corrosion behaviour. Characterisation of as-deposited coating confirms a dense, nanocrystalline B1-type TaC structure with excellent adhesion and negligible interdiffusion at the coating-substrate interface. In situ transmission electron microscopy with heavy-ion irradiation up to ~8 dpa reveals exceptional radiation resistance, with no evidence of amorphization, phase decomposition, or radiation-induced segregation. Limited grain coarsening and the formation of Xe bubbles (~2 nm) were observed. Electrochemical measurements in borated aqueous media demonstrated rapid passivation, very low corrosion current densities, and high impedance over prolonged exposure. These results identify nanostructured TaC as a robust and scalable candidate coating for accident-tolerant fuel cladding systems.

## Introduction

Zirconium (Zr) alloys are the materials of choice for nuclear fuel rods in Light-Water Reactors (LWRs) due to their neutron transparency (i.e., low thermal neutron absorption cross-section) combined with their thermal and mechanical stability^1^. The surface of the Zr rods oxidises almost immediately in the ambient environment due to its reactivity with oxygen. This oxide layer inhibits subsequent oxidation, leading to self-passivation of the Zr surface, which is stable at standard operation temperatures of a reactor^2,3^. In the case of a loss-of-coolant accident (known as LOCA), the reactor’s core reaches temperatures where continuous oxidation of Zr takes place, leading to the consumption of oxygen from the water in the reactor's core and the formation of hydrogen gas, which can, in the worst-case scenario, cause an explosion and meltdown as happened during the nuclear accident in Fukushima-Daiichi^4^. This event has dramatically demonstrated that further safety improvements are needed, leading to major research efforts on accident-tolerant fuel (ATF) claddings^5,6^.

One potential route to increase the safety in operation and under accident conditions is to apply a protective coating to zirconium-based fuel rods. Metallic Cr is a candidate coating material due to its high oxidation and radiation resistance^7,8^. Transition metal nitrides and MAX phase coatings are currently also considered as candidates for ATF claddings. Titanium nitride (TiN) based coatings have been studied to some extent as potential protective coatings for fuel rods^9–12^. However, Tunes et al. ^9^ have recently shown that TiN-based coatings may not fulfil the requirements for nuclear applications since they suffer from radiation-induced segregation (RIS), resulting in Ti-rich areas which may act as oxidation sites similar to pure Zr. Cr segregations were observed in Cr_2_AlC upon Xe radiation^13^, and Wang et al. ^14^ have shown that inert gas bubbles form at the coating (V_2_AlC) substrate (Zr) interface, which could lead to a diminished adhesion of the coating upon irradiation. Either during manufacturing or in operation in a nuclear reactor, pure Cr coatings can suffer oxidation reactions leading to the formation of highly toxic compounds – such as hexavalent chromium – posing a health concern to workers and to the environment^15^.

Tantalum carbide (TaC) potentially possesses a combination of attractive properties for ATF systems in Pressurized Water Reactors (PWR), such as high melting point and high thermal stability^16^, high hardness and fracture toughness^17,18^, low coefficient of friction and high wear resistance^19^, and high corrosion/oxidation resistance. Ta features a rather high capture cross-section for thermal neutrons, while C is at the lower end of the cross-section spectrum^20,21^, and, therefore, TaC should be a balanced compromise in terms of neutron absorption. This work aims to provide an in-depth understanding of the potential of TaC as ATF from the point of view of deposition feasibility/compatibility and irradiation resistance. We have deposited a nanostructured TaC ceramic coating on ZIRLO™ fuel cladding alloy as a substrate. ZIRLO™ is a Westinghouse Electric Corporation patented Zr alloy with 0.5-2.0 Nb (niobium), 0.7-01.5 Sn (tin), 0.07-0.28 Fe+Ni+Cr (iron, nickel, chromium) in wt.% and up to 200 ppm C^22^. The new ATF system (i.e., TaC onto ZIRLO™) was then subjected to in situ heavy ion irradiation in a transmission electron microscope (TEM) to investigate potential morphological changes caused by radiation damage, such as grain growth, radiation-induced precipitation or segregation (RIP or RIS), and inert gas bubble formation.

## Results

### Characterisation of the as-deposited coating

**Fig. 1:**
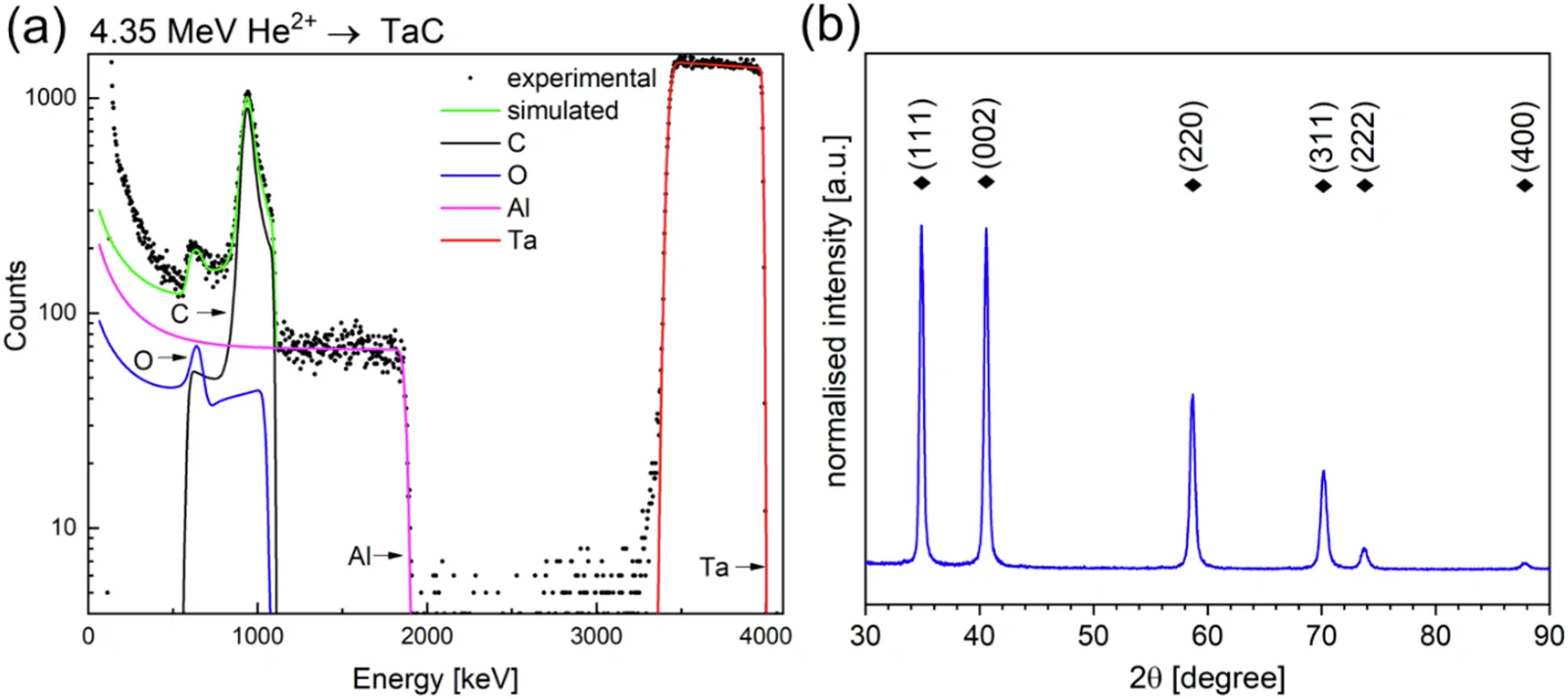
IBA of the as-deposited TaC nanoceramic coating. The fitted EBS spectra from compositional analysis is shown in **a**. The GI-XRD is shown in **b** with black diamond-shaped markers indicate the peak position for the NaCl-type structure with a cell parameter of a *=* 4.449 Å.

The as-deposited TaC coating was primarily characterised using IBA methods, GI-XRD, STEM, STEM-EDX, and TEM. The results of the composition and structure analysis of the TaC coating are summarised in Table 1. The composition was determined using a combination of IBA methods, namely EBS for the metal-to-carbon ratio and ToF-E-ERDA for the oxygen content. The Ta/C ratio was preferred to be extracted from EBS rather than from ERDA, due to the complicated multiple-scattering occurring for the latter method. The experimental EBS spectra, as well as the fits obtained from SIMNRA, are shown in Fig. 1a. A distinct plateau is seen around 3350–4000 keV in the EBS spectrum (Fig. 1a), corresponding to the signal of Ta, whereas the peak around 560 to 1120 keV corresponds to the C signal. The Ta to C ratio is deduced to 1.05, and therefore the TaC coating is stoichiometric within the Poisson error on the measurement. The Al-signal originates from the Al_2_O_3_ substrate, and the O signal can be either from the substrate or the coating. Additional ToF-E-ERDA measurements revealed that the impurity level of oxygen within the coating was below 1 at.%. The GI-XRD pattern in Fig. 1b shows all allowed reflections for a B1 NaCl-type structure, and no indications of the Ta_2_C phase are observed. The cell parameter was determined (by averaging all the reflections) to be a = 4.449 Å, which is in good agreement with the values obtained by ab initio density functional theory (DFT) calculations **a**_DFT_ = 4.48 Å and values for TaC thin films deposited by magnetron sputtering^23,24^.

**Table 1:** Elemental composition of the as-deposited ceramic coating

	Elemental Composition [at.%]			
	Ta	C	Crystal structure	a [Å]
TaC	51	49	NaCl (B1)	4.449(2)

**Fig. 2:**
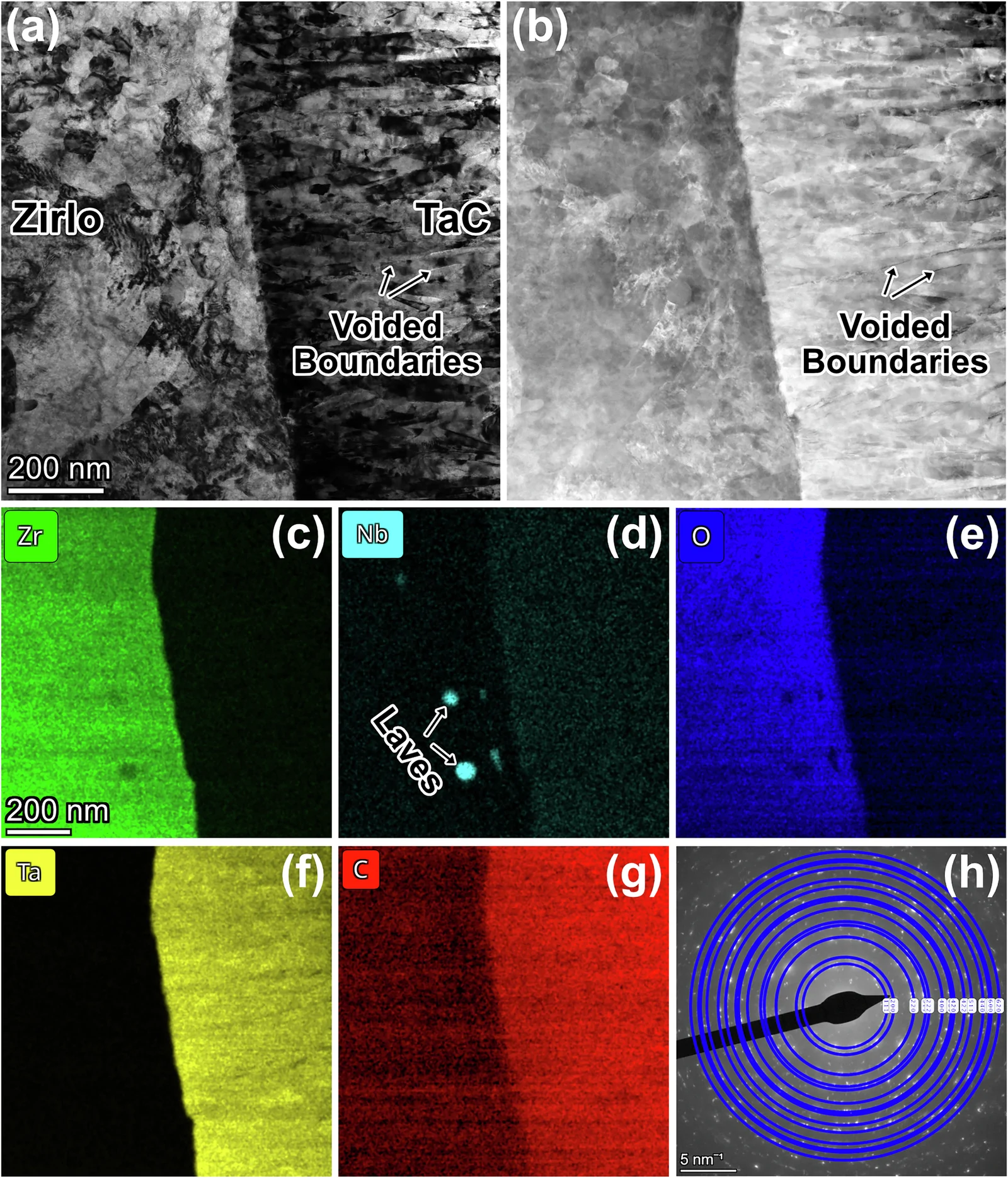
Pre-irradiation STEM characterisation of the TaC nanoceramic coating. **a** BF-STEM, and **b** HAADF images of the entire film cross-section. **c**–**g** STEM-EDX maps of the film-substrate interface. **h** SAED pattern indexed with data from the ICSD database^29^.

Figure 2 presents the STEM analysis of the TaC-coated ZIRLO^TM^ in the as-deposited state. The BF-STEM images in Fig. 2a shows that film exhibits a columnar microstructure where the columns consist of several nanocrystalline grains. Neither BF-STEM nor HAADF micrographs clearly reveal the presence of a native oxide layer of the Zr-substrate, but this is assumed to be ~5 nm thick. The columnar boundaries are separated by so-called voided regions – see arrows in Fig. 2b. Such a growth mode is reported for NbC^25^ and TaW-rich multicomponent carbide films^26^, and is associated with zone 1 growth^27^. The STEM-EDX maps – Fig. 2c–g – show that no interdiffusion between the Zr-substrate and the TaC film occurred, pointing to no elemental segregation or secondary particle formation within the interfacial layer. This indicates almost perfect adhesion between the film and the substrate. The STEM-EDX maps also revealed Nb-rich secondary phases in the ZIRLO™ substrate. The accurate chemical composition and structure of second-phase particles (SPPs) for ZIRLO™ is debated, but such clusters indicate the presence of either a C14 Laves phase with a composition close to ZrNb_2_ or another intermetallic phase^28^. The SAED pattern exhibits circular elongation of the diffraction spots, which are consistent with the polycrystalline microstructure, and the diffraction rings are indexed using the B1 NaCl-type structure^29^.

### In situ TEM with heavy-ion irradiation

**Fig. 3:**
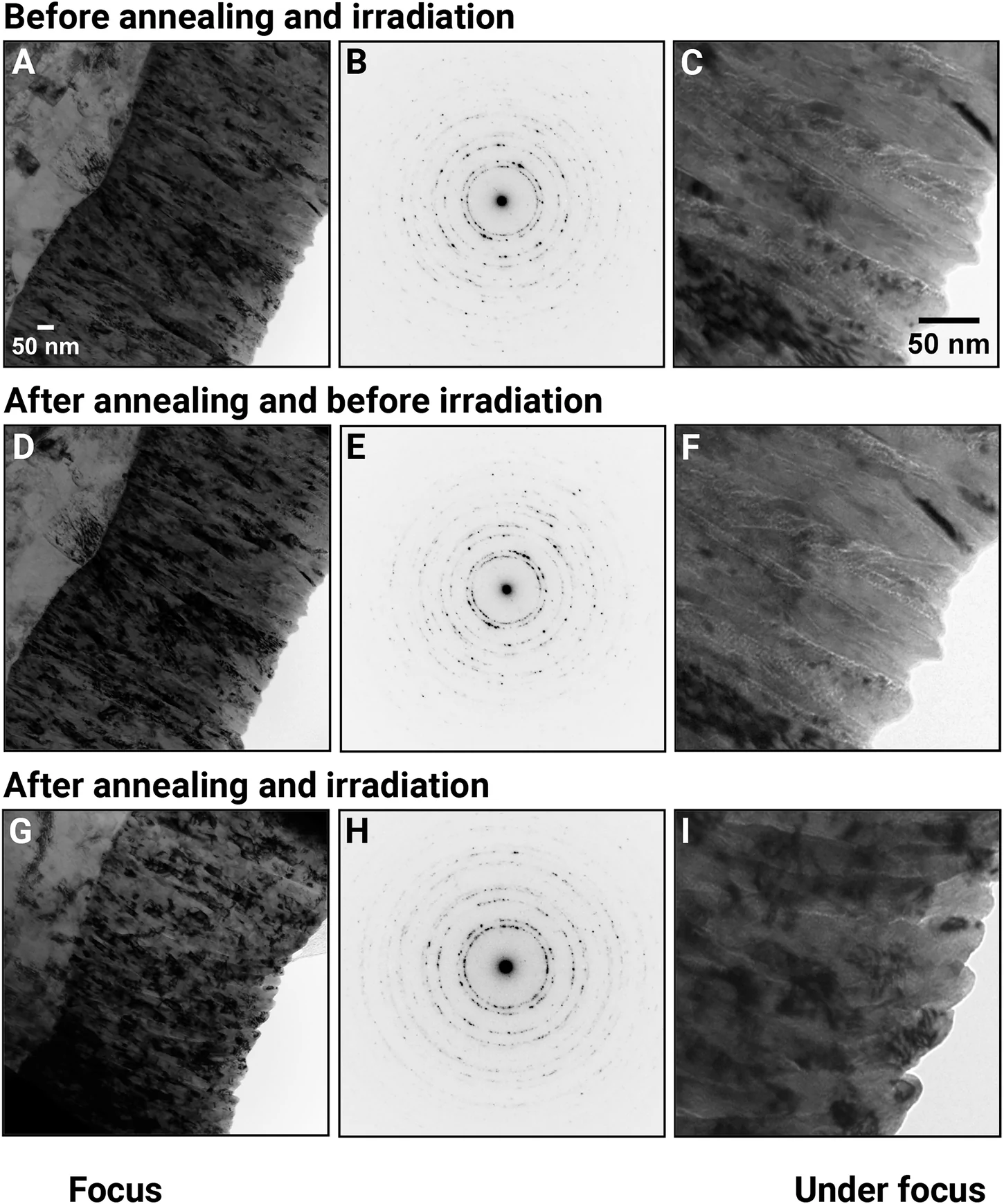
Irradiation behaviour monitored with in situ TEM. BF-TEM images of **A** as-deposited, **D** annealed, and **G** annealed and irradiated. SAED pattern of **B** as-deposited, **E** annealed, and **H** annealed and irradiated. SAED of **B** as-deposited, **E** annealed, and **H** annealed and irradiated. Underfocus BF-TEM images of **C** as-deposited, **F** annealed, and **I** annealed and irradiated. Note: the scale bar in **A** also applied to **D** and **G**; the scale bar in **C** also applies to **F** and **I**.

The microstructural changes upon annealing and Xe irradiation were studied using in situ TEM, and the recorded BF-TEM images and SAED patterns are shown in the micrographs in Fig. 3a–i. A comparison of the BF-TEM images – Fig. 3a, d – respectively of the as-deposited sample and the sample annealed at 300 °C for 30 min revealed that the columnar width of the nanostructured microstructure remained constant at ~ 20 nm, indicating good resistance to thermal annealing temperatures of relevance for LWRs. Fig. 3g shows the microstructure of the TaC coating after an average irradiation dose of 8 dpa: a modest grain growth to a columnar width of ~30 nm was observed. The SAED patterns in Fig. 3b, e, h reveal that no phase decomposition, precipitation, and/or amorphization that may have occurred due to either annealing or energetic particle irradiation, indicating an extraordinarily high stability within the simulated conditions of LWRs.

**Fig. 4:**
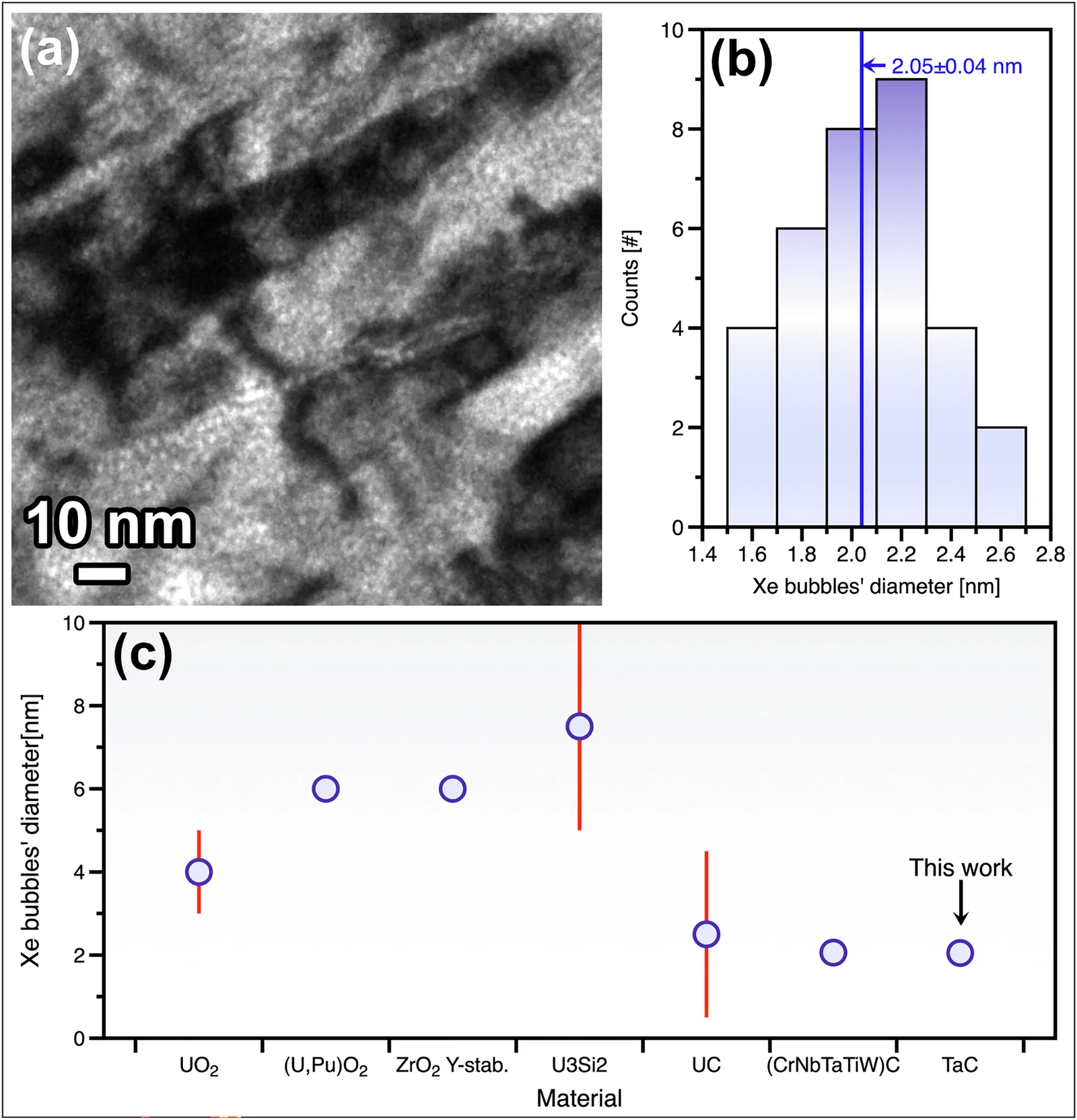
Quantification of Xe nanobubbles. **a** BF-TEM image of the irradiated material showing the Xe bubbles. **b** Histogram of the average size of the observed Xe bubbles after irradiation. Collection of data in **c** from literature shows that the Xe bubbles in our present TaC are as small as complex HECs such as the (CrNbTaTiW)C^37,38^ and much smaller than Xe bubbles detected in conventional nuclear ceramics. Data in **c** was extracted from Tunes et al. ^37^.

The major radiation damage type of defect detected in the nanostructured TaC subjected to irradiation was the formation of nanometre-sized Xe bubbles. The BF-TEM image in Fig. 4a shows a detailed view of the irradiated microstructure at 8 dpa, which clearly shows the presence of transgranular Xe bubbles, which exhibit a white-bright appearance when imaged under an underfocused condition. The average bubble size was estimated to be ~2.1 nm at 8 dpa, and the entire bubble diameter distribution as measured is presented in the histogram in Fig.4b. A compilation of data on Xe bubbles in many nuclear ceramics as well as our previous HEC is shown in Fig. 4c, where it is possible to note that the TaC exhibit Xe bubble sizes as small as the HEC, pointing its high-radiation tolerance.

### Corrosion behaviour in simulated reactor conditions

**Fig. 5:**
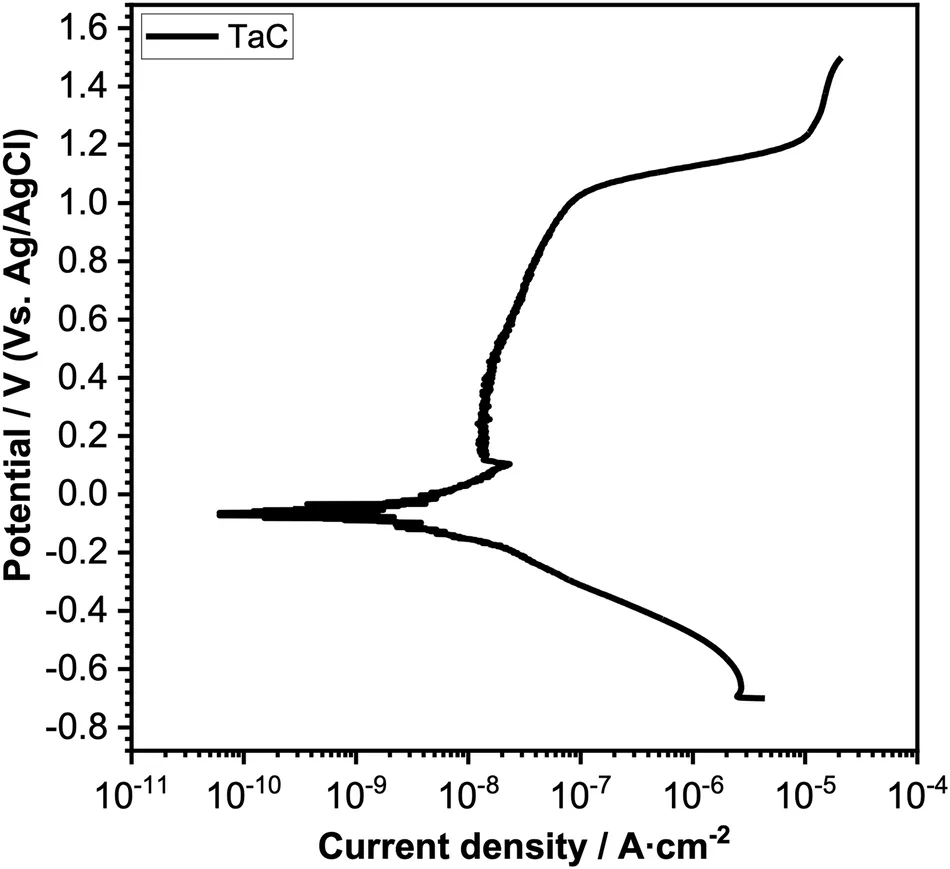
Potentiodynamic test. Polarisation curve for the TaC nanoceramic coating on Al_2_O_3_ substrate.

The potentiodynamic polarization curves obtained for the TaC coatings on Al_2_O_3_ substrates (presented in Fig. 5) reveal a characteristic response of a passive ceramic-coated zirconium alloy in aqueous borated media, consistent with the behaviour reported for TaC-based coatings in the literature^30–32^. At low anodic overpotentials, the material initially exhibits an active dissolution region, but this stage is narrow and quickly transitions into a current-limited plateau, indicating the formation of a stable passive layer on the TaC surface. From this plateau up to approximately 1.2 V vs. (Reference electrode), the coating maintains a quasi-stationary current, after which a marked increase in current density is observed, suggesting the onset of transpassive oxidation as also observed in other TaC-containing systems exposed to aqueous electrolytes^33^.

The corrosion potential and corrosion current density determined by Tafel extrapolation were –63 mV vs. (Reference electrode) and 3.2 × 10⁻⁹ A·cm⁻², respectively, values that reflect an excellent resistance to electrochemical degradation. These very low current densities are comparable to or smaller than those reported for engineered Ta-based coatings designed for harsh environments, such as TaN/TaC/C multilayers or Ta-containing amorphous alloys, where i_corr_ values below 10⁻⁸ A·cm⁻² are commonly achieved due to the stability of Ta-oxide passive films^34,35^. Similarly, TaC coatings deposited on metallic biomaterials also show rapid passivation and significantly reduced corrosion rates compared to their substrates, attributed to dense nanocrystalline structures and chemically stable passive films^32^.

When comparing with other coating systems two aspects stand out: (i) unlike Ti- or Ni-based coated substrates, which often display more extended active-passive transitions or susceptibility to localized attack, the TaC ATF coating system on Al_2_O_3_ substrate shows immediate stabilization of anodic current, indicating an efficient barrier effect; and (ii) the low corrosion current density (i_*corr*_) and stable passive plateau are consistent with findings for TaC-reinforced or Ta-rich multilayer coatings used in highly corrosive media (e.g., NaCl, H₂SO₄, Ringer’s solution), where Ta-oxide formation governs corrosion resistance^32,36^. This agreement reinforces that the sputtered TaC nanostructure on Al_2_O_3_ substrates effectively forms a protective, self-limiting passive layer as observed in other TaC-based protective systems, independent of application context (nuclear, biomedical, or tribological environments).

**Fig. 6:**
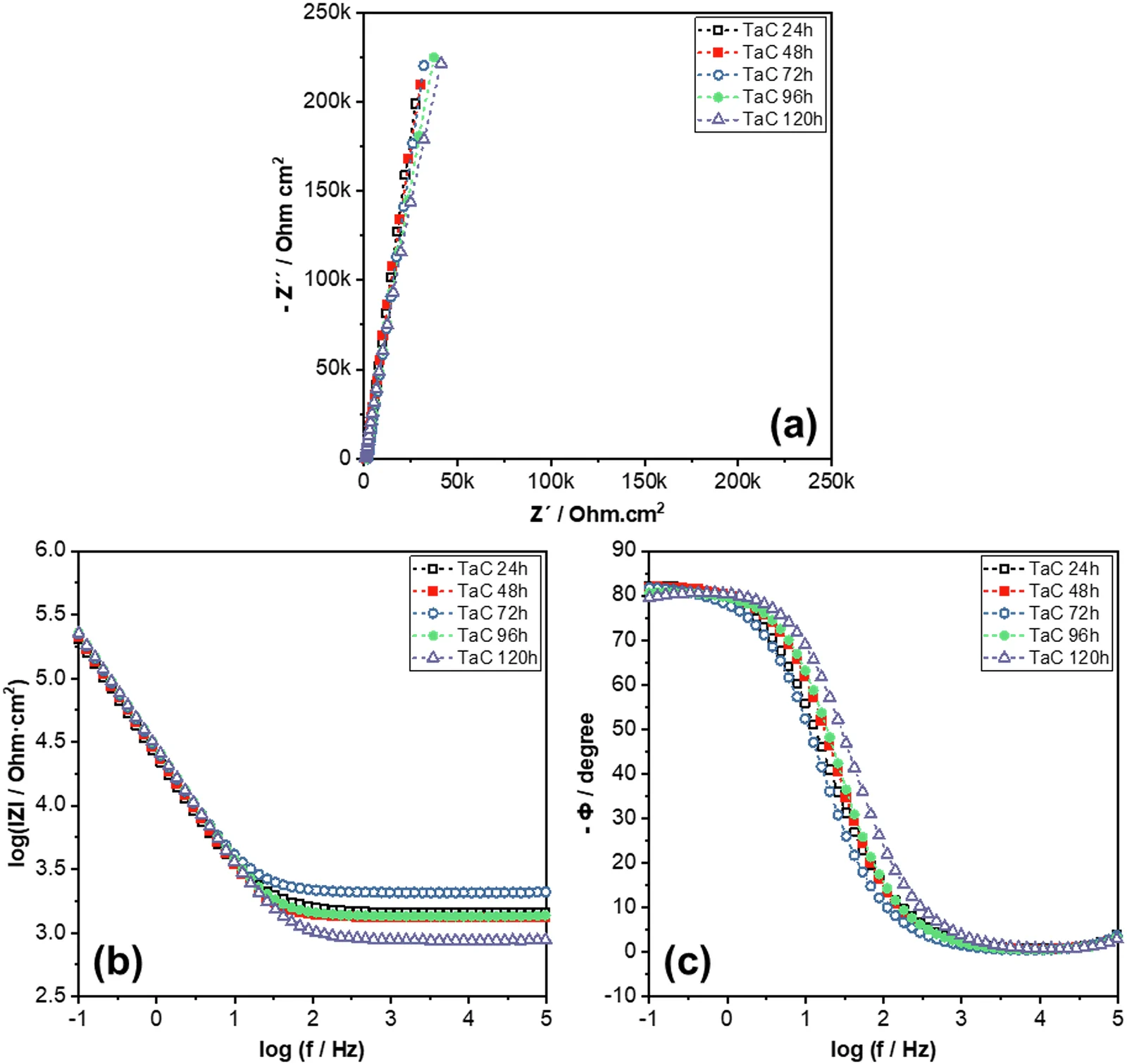
Corrosion behaviour of TaC nanoceramic coating. Nyquist (**a**) and Bode (**b**, **c**) diagrams of the TaC coating on Al_2_O_3_ substrate after 24, 48, 72, 96, and 120 h of exposure.

**Fig. 7:**
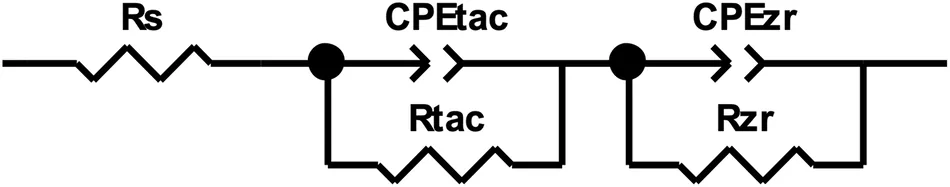
Electrochemistry of TaC nanoceramic coating. An electrical equivalent circuit is used to fit the EIS data obtained via testing of the TaC coating.

The EIS spectra of the TaC coatings presented in Fig. 6 – Nyquist 6(a) and Bode 6(b, c) – show a single, wide capacitive time constant across mid–low frequencies and very large impedance modulus values (>10⁵ Ohm·cm^2^) for all exposure times, which is consistent with a dense, electronically blocking/passivating surface formed by the TaC film. The data were fitted with the equivalent circuit shown in Fig. 7, where CPE_tac_ and R_tac_ represent the TaC film response and CPE_zr_/R_zr_ the substrate/oxide response beneath defects. The fitted CPE exponents (n_tac_ ≈ 0.91–0.96; n_zr_ = 1.0) indicate a nearly ideal capacitive behaviour of the coating/oxide system, and the fitted curves match the experimental points well (crosses vs open symbols), confirming the chosen physical model. Similar high-impedance, rapid passivation behaviour has been reported for dense TaC coatings and Ta-rich multilayers, where Ta-oxide formation provides a stable barrier^32,35^.

Quantitatively, the fit parameters shown in Table 2 indicates a gradual decrease of both film and substrate resistances with immersion time, but a clear maintenance of high protection after long exposure: R_tac_ falls from ~9.8 × 10⁶ Ohm·cm² at 24 h to ~4.2 × 10⁶ Ohm·cm² at 120 h, and R_zr_ from ~2.6 × 10³ Ohm·cm² to ~8.8 × 10² Ohm·cm² over the same interval. This modest decay suggests some penetration/evolution at defects/pores, but overall excellent electrochemical stability of the TaC barrier, a behaviour in line with other works that report >90% protection efficiency and stable Ta-oxide passivation for TaC/Ta systems and TaC-based multilayers^32,36^.

**Table 2:** Results of the fitting of the EIS diagrams (Fig. 7) using the equivalent electrical circuit shown in Fig. 8 for the TaC coating on Al_2_O_3_ substrate

Test time (h)	R_s_ Ohm·cm^2^	CPE_tac_ F cm^−2^·s^2(n−1)^	n_tac_	R_tac_ Ohm·cm^2^	CPE_zr_ F cm^−2^·s^2(n−1)^	n_zr_	R_zr_ Ohm·cm^2^
24	4.93E−04	7.81E−06	0.96	9.76E + 06	1.24E−11	1	2.56E + 03
48	7.33E−04	7.29E−06	0.94	7.58E + 06	4.69E−11	1	2.49E + 03
72	1.07E−03	6.89E−06	0.92	5.17E + 06	6.46E−11	1	2.08E + 03
96	2.68E−03	6.65E−06	0.92	4.41E + 06	6.51E−11	1	1.35E + 03
120	3.36E−03	6.56E−06	0.91	4.24E + 06	9.33E−11	1	8.77E + 02

In summary, the corrosion behaviour of the TaC ATF system shows the following: (i) the TaC coating produces a dominant capacitive response and very large impedance throughout 120 h, (ii) the single electric-equivalent circuit used appropriately describes the ATF system and the CPE parameters point to a compact, near-ideal passive film, and (iii) although R_tac_ and R_zr_ decrease slightly with time, the coating preserves high corrosion resistance after prolonged immersion, confirming its effectiveness as an electrochemical barrier.

## Discussion

In this present work, the results obtained with the synthesis and irradiation testing of the nanostructured TaC ceramic coating should be analysed within the context of our previous research with high-entropy ceramics (HEC)^37,38^. We have previously synthesised and irradiated a new (CrNbTaTiW)C HEC with identical irradiation conditions, but up to an average dose of 10 dpa^37^. We found that the HEC coating was extremely tolerant to irradiation, exhibiting neither amorphisation nor precipitation nor any other form of phase decomposition or elemental segregation. The major obstacle to the use of highly complex materials such as the (CrNbTaTiW)C HEC in nuclear systems lies in their large-scale fabrication^37^. Current powder-metallurgical methods preclude the synthesis of HECs in large quantities and geometries. This is not the case for chemically simpler ceramic systems such as the Ta–C. Nevertheless, a major outcome of our previous work was that nanostructure HECs are highly radiation tolerant, given the presence of many nanometre-sized grains that act as strong sinks for irradiation-induced point defects^37^.

The TaC coating reported in this work also exhibited excellent radiation tolerance under the applied irradiation conditions (average dose of ~8 dpa, local peak dose of ~20 dpa). Throughout the entire irradiation sequence, no evidence of amorphisation or phase transformation was detected, consistent with the high structural stability of the rock salt lattice and its strong covalent-metallic bonding character. Moreover, no RIS/RIP phenomena were observed, neither within the grains nor along interfaces. This behaviour contrasts with reports on other proposed ATF coating systems, including TiN-based materials^9^ and several MAX-phases^13^, where radiation triggers elemental redistribution, defect-assisted clustering, or nanobubble formation that can degrade the coating’s functionality. The absence of any such effects in TaC indicates a high resistance to irradiation-driven defect transport and solute mobility, suggesting that TaC maintains its microstructural integrity even under extreme displacement-damage conditions relevant for nuclear fuel operation. This result places the TaC nanoceramic coating with simplified chemistry at the same level of irradiation resistance as the chemically complex HECs previously tested^37,38^. We also point out that the chemical composition is not a strict factor in the design of radiation-tolerant transition metal carbide ceramics: rather, nanostructured microstructure and the rock-salt structure are herein confirmed as underlying facts behind the observed irradiation resistance.

A further noteworthy observation is the closure of pre-existing voided grain boundaries during irradiation. In as-deposited coatings, such boundaries may raise concerns regarding gas permeability or accelerated entry of corrosive species. Under irradiation, however, the collapse or densification of these boundaries is beneficial, as it effectively reduces open diffusion pathways and produces a more compact microstructure. This behaviour distinguishes TaC favourably from many oxide and nitride systems, which often experience irradiation-induced void swelling, boundary decohesion, or pore formation that compromise their barrier properties^39^. The irradiation-assisted healing effect of voided boundaries in TaC (i.e., nanograins acting as sinks for point defects induced by irradiation) enhances its suitability as an ATF coating, as it supports both structural robustness and long-term resistance against corrosive and oxidising environments.

To summarise, a TaC nanoceramic coating deposited onto ZIRLO™ fuel cladding by DC magnetron sputtering exhibited outstanding performance under light-water reactor–relevant conditions, combining excellent substrate adhesion, exceptional radiation tolerance up to ~8 dpa average dose with no amorphisation, phase decomposition, or radiation-induced segregation – only minor grain coarsening and nanometre-sized Xe bubbles – and robust corrosion resistance characterised by rapid passivation, negligible corrosion currents, and stable capacitive behaviour in borated aqueous media. These results collectively demonstrate that TaC nanoceramic is a highly promising accident-tolerant fuel cladding coating, leveraging both the intrinsic stability of the rock-salt lattice and its nanocrystalline microstructure to efficiently mitigate radiation damage whilst providing durable electrochemical protection of the underlying zirconium alloy. Corrosion test under operating conditions of a light water reactor requires specialized infrastructure (300 °C and 7–16 MPa) and is planned to confirm the enhanced corrosion. In this way, the PWR-simulated tests are beyond the scope of the present study.

Overall, the present results indicate that nanostructured thin film TaC constitutes a superior alternative to pure Cr-based coatings for accident-tolerant fuel claddings. In contrast to Cr coatings, which may suffer from oxidation-induced degradation and raise concerns related to the formation of toxic hexavalent chromium species, TaC offers excellent irradiation tolerance, microstructural stability, and corrosion resistance without such environmental or health drawbacks. Nevertheless, a highly stable ceramic coating onto ZIRLO™ can mitigate possible interdiffusion from the coating into the substrate. This is opposite to the processes of observed radiation-induced segregation of Cr into the zirconium fuel cladding and/or Zr into the Cr coating. The latter leads to deleterious degradation of the entire ATF system. Together with its high thermal stability and compatibility with scalable sputtering processes, these factors position TaC as a more robust and sustainable coating solution for advanced nuclear fuel cladding applications.

## Methods

### Synthesis of the TaC nanoceramic thin films

The films were synthesised by non-reactive DC-magnetron sputtering from Ta and C circular 2″ targets with a claimed purity of 99.9%. The chamber base pressure was below 3.8 × 10^−7 ^Pa. The ZIRLO™ and the single-crystal α-Al_2_O_3_ (001) substrates were placed on a rotating substrate holder and were pre-heated to 500 °C for one hour to minimise temperature gradients. A 42 sccm Ar gas flow rate was used to ignite an Ar^+^ plasma with a pressure of 0.6 Pa. A substrate bias of −100 V was applied.

### Ion beam analysis and Diffractometry

Elastic Backscattering Spectroscopy (EBS) experiments were performed at the Tandem Laboratory at Uppsala University 4.35 MeV He^2+^ as primary ions^40^. The advantage of EBS compared to Rutherford Backscattering Spectrometry (RBS) is that EBS also considers elastic scattering events and therefore C content can be better determined. The elastic scattering event ^12^C(^4^He,^4^He)^12^C featuring a cross-section resonance near an ion energy of 4.26 MeV^41^ was used to obtain the C fraction in the film. The measurements were carried out with the incident beam at 5° with respect to the normal surface, and the backscattering angle was 170°. The samples were wiggled within a 2° angle interval to avoid possible channelling effects. The SIMNRA software package was for data analysis^42^ using the cross sections from Sigma Calc to fit the C peak^43^.

Grazing incidence X-ray diffraction (GI-XRD) measurements with an incidence angle of 2° were carried out using a Philips MRD X’Pert diffractometer. The diffractometer was operated in a parallel beam geometry using a Göbel mirror on the primary side and a parallel plate collimator with a 0.27° divergence on the secondary side.

### Sample preparation for electron microscopy

Cross-section samples for TEM were prepared using a Zeiss Focused Ion Beam (FIB)/SEM Crossbeam 550 with Ga Ion-Sculptor gun and an Omniprobe 200. The obtained lamellas were attached to a Cu lift-out grid and thinned with a 30 kV Ga-ion beam, finishing with a polishing step using a 2 kV ion beam to mitigate surface damages and ion implantation. Transmission electron microscopy (TEM) analysis was carried out using a FEI Talos F200X STEM operating a field emission gun with the energy set to 200 keV. Post-irradiation scanning transmission electron microscopy (STEM) characterisation was performed via the Super-X Energy-Dispersive X-ray (EDX) spectroscopy technique, High-Angle Annular Dark-Field (HAADF), and Bright-field TEM (BF-TEM), as well as Selected Area Electron Diffraction (SAED).

### In situ TEM ion irradiation

**Fig. 8:**
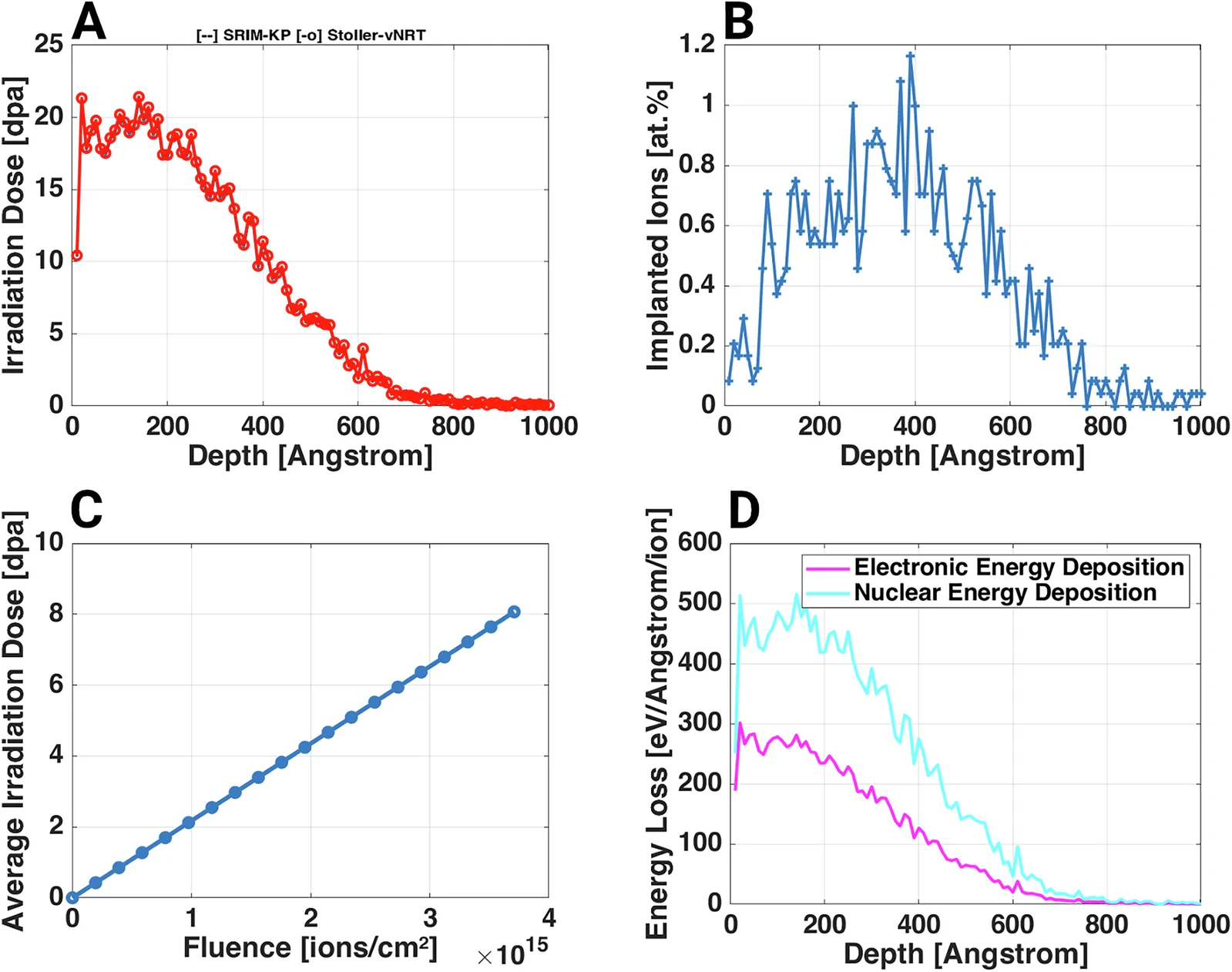
Radiation damage calculations with SRIM2013Pro. Showing the calculations as a function of depth for **A** displacements-per-atom, **B** implanted ions, **C** the average irradiation dose (as a function of fluence), and **D** the energy loss.

The lamellas were irradiated with a 300 keV Xe^+^ ion beam with a flux of 6.2 × 10^12 ^ions cm^−2^ s^−1^ up to a fluence of 3.7 × 10^15 ^ions cm^−2^ at 300 °C (with a hold time of 30 min), using the MIAMI-2 system at the University of Huddersfield^44^. The angle between the ion and the electron beam is 18.7°, and therefore the angle between the sample cross-section and the ion beam is 71.3°. SRIM-2013Pro was used to convert fluence to dpa^45^ assuming a lamella thickness of 100 nm and a density of 14.3 g cm^−3^. The calculation mode was the Kinchin-Pease (KP, quick-damage). Under these conditions, the maximum fluence of our experiments corresponds to an average dose of 8 dpa and a peak dose of around 20 dpa. An average of 0.42 at.% of Xe has been implanted into the TaC. All SRIM calculations are presented in Fig. 8. The 300 keV Xe^+^ ions lead to a considerable level of implantation for the electron-transparent lamella, and this fact was used to investigate Xe retention (fission gas within the U decay chain).

### Electrochemical tests

Electrochemical measurements were performed using a Teflon laboratory-made three-electrode electrochemical cell connected to an Autolab PGSTAT302N potentiostat/galvanostat (Metrohm Instruments). In these experiments, the coating samples (tantalum carbide TaC on Al_2_O_3_) were used as a working electrode, together with an Ag/AgCl (3 M NaCl) reference electrode (205 mV vs. SHE) and a Pt wire as the counter electrode. In this setup, the connection of the coating to the potentiostat was made using copper tape positioned outside the electrochemical cell. The exposed area was 0.2 cm². All experiments were carried out at room temperature and a 10 mM borated water solution as the electrolyte, since borate salts are used to thermalize neutrons in light water reactors^46^.

Before each experiment, the open-circuit potential (OCP) was monitored for 120 min to allow stabilization of the electrode–electrolyte interface. The electrolyte volume used in the cell was fixed at 4 mL, and each measurement was repeated at least three times to ensure reproducibility.

Electrochemical Impedance Spectroscopy (EIS) measurements were conducted at the stabilized OCP by applying a sinusoidal voltage perturbation of 10 mV. The frequency range spanned from 10⁻¹ Hz to 10⁵ Hz. The impedance spectra were fitted and interpreted using ZView 4, employing equivalent-circuit models selected according to the electrochemical behaviour of the as-deposited TaC.

Potentiodynamic polarization curves were acquired after OCP stabilization. The potential was swept from −700 to 1500 mV vs Ag/AgCl (3 M NaCl) at a scan rate of 1 mV s⁻¹. Corrosion parameters, including the corrosion potential (Ecorr) and corrosion current density (icorr), were extracted by Tafel extrapolation.

## Data Availability

The datasets generated and/or analyzed during the current study are publicly available in the Mendeley Dataset: Fritze, Stefan; Osinger, Barbara; Ström, Petter; Eklund, Per; Prada Ramirez, Oscar M.; Vishnyakov, Vladimir; Tunes, Matheus A (2026), “Data for: Exceptional irradiation and corrosion resistances of a TaC nanoceramic coating deposited onto ZIRLO™ fuel cladding”, Mendeley Data, V1, doi: 10.17632/sd3w232xx9.1
